# Effect of Enhanced ADAS Camera Capability on Traffic State Estimation

**DOI:** 10.3390/s21061996

**Published:** 2021-03-12

**Authors:** Hoe Kyoung Kim, Younshik Chung, Minjeong Kim

**Affiliations:** 1 Department of Urban Planning and Engineering, Dong-A University, Busan 49315, Korea; hoekim@dau.ac.kr (H.K.K.); kmlalswjd@hanmail.net (M.K.); 2 Department of Urban Planning and Engineering, Yeungnam University, Gyeungsan 38541, Korea

**Keywords:** advanced driver assistance system (ADAS), image-based vehicle identification, market penetration rate, microscopic traffic simulation, normalized root mean square error, probe vehicle

## Abstract

Traffic flow data, such as flow, density and speed, are crucial for transportation planning and traffic system operation. Recently, a novel traffic state estimating method was proposed using the distance to a leading vehicle measured by an advanced driver assistance system (ADAS) camera. This study examined the effect of an ADAS camera with enhanced capabilities on traffic state estimation using image-based vehicle identification technology. Considering the realistic distance error of the ADAS camera from the field experiment, a microscopic simulation model, VISSIM, was employed with multiple underlying parameters such as the number of lanes, traffic demand, the penetration rate of ADAS vehicles and the spatiotemporal range of the estimation area. Although the enhanced functions of the ADAS camera did not affect the accuracy of the traffic state estimates significantly, the ADAS camera can be used for traffic state estimation. Furthermore, the vehicle identification distance of the ADAS camera and traffic conditions with more lanes did not always ensure better accuracy of the estimates. Instead, it is recommended that transportation planners and traffic engineering practitioners carefully select the relevant parameters and their range to ensure a certain level of accuracy for traffic state estimates that suit their purposes.

## 1. Introduction

Traffic flow data, such as flow, density and speed, are used as the primary data for transportation planning and traffic operation [1]. Diagnosing current traffic states and predicting future traffic conditions requires a fast and extensive data-collection process. In general, traffic flow data are directly collected or estimated by detectors such as Automatic Vehicle Classification (AVC) systems, Vehicle Detection Systems (VDSs) and Automatic Vehicle Identification (AVI) systems, installed at specific locations. However, there are spatial limitations in acquiring detailed and wide-ranging traffic information simultaneously in an entire network and there is a high cost for installing, operating and maintaining stationary detectors. Therefore, it is very difficult to collect traffic flow data on all the roadways across a country [2,3,4,5,6].

Efforts have been made to overcome the spatial limitations of stationary detectors and some researchers have suggested a method of estimating traffic state using probe vehicles [3,5,7]. This method estimates the density with the distance to the leading vehicle measured by an Advanced Driver Assistant System (ADAS) camera, which is mounted on a vehicle and is equipped with functions such as Lane Departure Warning (LDW), Forward Collision Warning (FCW) and Autonomous Emergency Braking (AEB) to improve safety and convenience [8,9].

The National Highway Traffic Safety Administration (NHTSA) of the U.S. Department of Transportation reported that 20 automakers have committed to equipping all new passenger vehicles with low-speed FCW and AEB by September 2022 [10]. The European Commission has recently revised its general safety regulations and mandated that LDW and AEB be installed in light-duty vehicles by 2022 [11]. South Korean legislation required ADAS devices to be installed on commercial vehicles with lengths and weights that exceed 9 m and 20 tons in 2017 [12]. Therefore, the applicability of image-based vehicle identification technology linked with ADAS cameras is expanding the opportunities for traffic state estimation.

Unlike other studies on traffic state estimation with ADAS cameras, this study derives more realistic estimates by analyzing the experimental and theoretical distance error of an ADAS camera and reflecting it in a microscopic simulation model, VISSIM. This study also evaluates the accuracy of traffic state estimates (i.e., traffic volume, speed and density) concerning the number of lanes, traffic demand, market penetration rate (MPR) of ADAS vehicles and the spatiotemporal range of the estimation area. The focus is on the enhanced capabilities of the ADAS camera, including the identification distance and vehicle identification in the rear (e.g., through a backup camera for safety) and in adjacent lanes.

## 2. Related Works

Research associated with ADAS can be categorized into safety and traffic state estimation studies and is conducted mainly using simulation methods. On the other hand, most research efforts on ADAS have been limited to traffic safety-related studies because the ADAS device aims to ensure safety by generating an alarm that makes the driver react in the event of an accident with surrounding vehicles [13] or by letting the vehicle itself respond [14,15,16].

From the perspective of traffic safety, Jeong and Oh [17] concluded that LDW and AEB reduce frontal collision accidents by 10–14% and 50%, respectively. Sugimoto and Sauer [18] showed that an AEB system could reduce the number of collisions with a leading vehicle by 38% and the probability of death by 44%. Lyu et al. [19] revealed that drivers had a much higher acceptance of the FCW functions than the LDW functions. Louwerse and Hoogendoorn [20] concluded that ADAS can decrease the total number of accidents by 4% to 19% on non-motorway networks. Davidse [21] suggested that ADAS should be developed to improve the safety of older drivers.

Previous studies on traffic state estimations, based on multiple types of sensors and emerging technologies, dealt with the position estimation of an object by exploiting narrowband broadcast radio signals [22] to estimate and predict the traffic flow characteristics scalable to the urban level [23,24,25]. Image-based vehicle identification technology coupled with ADAS cameras was introduced recently for traffic state estimation [1,4,26]. The speed and volume can be estimated relatively easily using stationary detectors installed at fixed locations, but it is challenging to estimate the density of vehicles distributed in a certain range of traffic networks, which is an effective measure of uninterrupted traffic flow [27,28]. Table 1 lists the underlying differences between the two traffic state estimation methods.

Seo et al. [2] established a methodology to estimate fundamental diagrams using probe vehicle data. Hoogendoorn and Minderhoud [29] reported that autonomous intelligent cruise control (AICC) had a positive effect on bottleneck capacity. Seo and Kusakabe [7,30] developed and validated a method that estimates the traffic state based on the observed spacing and position data of probe vehicles.

**Figure 1:**
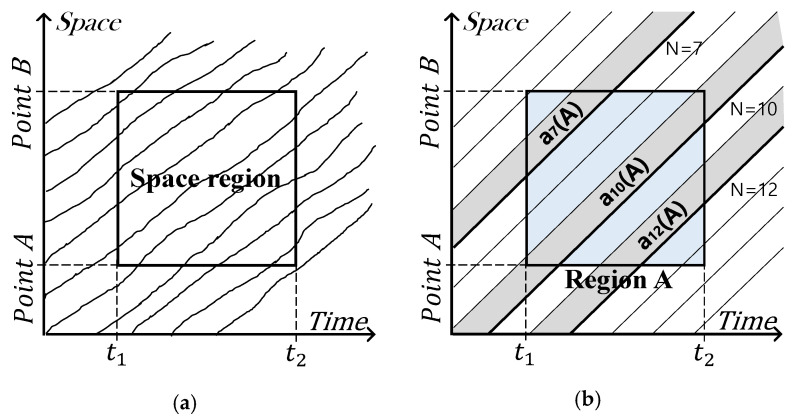
Density estimation method using vehicle trajectories: (**a**) Edie’s method [31]; (**b**) Seo et al.’s method [3].

Edie [31] proposed a method of estimating density based on the relationship between a specific spatiotemporal estimation area and the travel time and distance derived from the trajectory information of all vehicles traversing through the estimation area. Seo et al. [3], Seo et al. [5] and Seo and Kusakabe [7] basically used Edie’s method [31], but they used the estimation area created by extending the headway formed between the ADAS vehicle and the leading vehicle along the time axis rather than the entire spatiotemporal estimation area, as shown in Figure 1 and Equations (1)–(3).

(1) $$\hat{q}\left(A\right)=\frac{\sum_{n\in P\left(A\right)}^{\;}d_{n}\left(A\right)}{\sum_{n\in P\left(A\right)}^{\;}\left|a_{n}\left(A\right)\right|}$$

(2) $$\hat{u}\left(A\right)=\frac{\sum_{n\in P\left(A\right)}^{\;}d_{n}\left(A\right)}{\sum_{n\in P\left(A\right)}^{\;}\left|t_{n}\left(A\right)\right|}$$

(3) $$\hat{k}\left(A\right)=\frac{\sum_{n\in P\left(A\right)}^{\;}t_{n}\left(A\right)}{\sum_{n\in P\left(A\right)}^{\;}\left|a_{n}\left(A\right)\right|}$$

where:

$\hat{q}\left(A\right),\hat{k}\left(A\right),\hat{u}\left(A\right):$ flow, density and speed estimators for region A
P(A): set of all probe vehicles in region A
$a_{n}\left(A\right):$ time-space region between vehicle *n* and a leading vehicle in region A
$d_{n}\left(A\right):$ distance traveled by vehicle *n* in region A
$t_{n}\left(A\right):$ time spent by vehicle *n* in region A

Simulation techniques are mostly used for analysis of the effects of ADAS devices on traffic safety and traffic state estimation instead of reproducing traffic situations in the real world [32]. For instance, Lundgren and Tapani [33] and Louwerse and Hoogendoorn [20] quantified the impacts of ADAS on traffic safety through a microscopic simulation model. Detering and Schnieder [34], Golias et al. [35] and Olstam and Elyasi-Pour [36] used traffic simulation models to study the impact of ADAS on traffic flow. Hoogendoorn and Minderhoud [37] and Hoogendoorn and Minderhoud [29] addressed the effects of ADAS on efficiency, accuracy, driving comfort and safety with a microscopic simulation model. Tapani [38] and Massow and Radusch [39] proposed novel approaches to estimate the impact of ADAS on a traffic system, such as a two-step methodology and analysis framework for cooperative ADAS under a traffic simulation environment.

The main difference between this study and previous research is that it takes into account ADAS vehicles as a new traffic data-collection system rather than a means to improve traffic safety and evaluate its performance. The studies by Seo et al. [3], Seo et al. [5], and Seo and Kusakabe [7] lacked an understanding of the distance error between the ADAS vehicle and the leading vehicle and did not sufficiently evaluate the effect of varying traffic demand and MPR of ADAS vehicles. However, this study attains more realistic estimation results by investigating the experimental and theoretical distance error of the image-based vehicle identification technology with an ADAS camera from field experiments. A microscopic simulation model, VISSIM, was used to investigate the effect on the traffic state estimates of numerous parameters, such as enhanced ADAS camera capability (vehicle identification distance and vehicle identification in the rear and adjacent lanes), the road geometry (the number of lanes), traffic demand (level of service (LOS)), the MPR of ADAS vehicles and the spatiotemporal range of the estimation area.

## 3. Research Methodology

VISSIM is a stochastic, microscopic, time-step and behavior-based model [40] and is one of the most widely accepted analytical tools in traffic engineering research, which is a pivotal role in this study. Its user interface, VISSIM Component Object Model (COM), is capable of not only accessing and controlling underlying objects such as vehicles and traffic signals, but also expanding the research scope that cannot be implanted in the basic VISSIM model by synchronizing external algorithms and programs. Therefore, VISSIM can track the trajectories of individual vehicles to estimate the position of the leading vehicle and the distance to it from the ADAS vehicle.

Equations (4)–(6) are methods for estimating traffic flows on uninterrupted flow roadways using an ADAS camera. Estimation of the traffic flow rate is based on fundamental traffic flow theory (4) and the speed estimate is the space mean speed of all ADAS vehicles traveling inside the estimation area (5). The travel time and distance of the ADAS vehicles can vary depending on the spatiotemporal definition of the estimation area. The density estimate can be obtained by averaging the distance to the leading vehicle collected by all ADAS vehicles traversing through the estimation area (6). Seo et al. [3] measured the distance to the leading vehicle every 15 s, but in this study, the distance is estimated more accurately by measuring it at every unit of simulation time (i.e., 1 s).

(4) $$\hat{q}\left(A\right)=\hat{u}\left(A\right)\times \hat{k}\left(A\right)$$

(5) $$\hat{u}\left(A\right)=\frac{\sum_{n\in P\left(A\right)}d_{n}\left(A\right)}{\sum_{n\in P\left(A\right)}t_{n}\left(A\right)}\times 3.6$$

(6) $$\hat{k}\left(A\right)=\frac{1000}{\frac{\sum_{n\in P\left(A\right)}\sum_{m\in P\left(n\right)}h_{m}\left(A\right)}{P\left(A\right)M}}$$

where:

P(n): set of all simulation time units of ADAS vehicle *n* in region A
$h_{m}\left(A\right):$ headway of ADAS vehicle *n* at the *m*th simulation time in region A
M: the average amount of simulation time spent by all ADAS vehicles to measure the distance to the leading vehicles in region A

The capability of the ADAS camera is one of the primary parameters affecting the accuracy of traffic state estimates for the uninterrupted traffic flow. This study defines its capabilities as vehicle distance identification and vehicle identification in the rear and adjacent lanes and a more accurate traffic state estimation is expected with fewer ADAS vehicles. According to the FCW performance standards of ADAS vehicles, the FCW function should be activated when the time to collision (TTC) between an ADAS vehicle and a leading vehicle is at least 2.4 s [41]. However, in order to induce a quicker response from drivers, it is necessary to set the FCW activation time to longer than 2.4 s. The distance between ADAS vehicles and leading vehicles required for an activation time of 3 or 4 s at 100 kph is approximately 100 m. 

The number of lanes, traffic demand, the MPR of ADAS vehicles and the spatiotemporal range of the estimation area are also important parameters influencing the accuracy of the traffic state estimates. Table 2 shows all relevant parameters used in this study and their ranges. A total of 324,000 scenarios were proposed with predefined parameters and 30 simulation runs were implemented for individual scenarios to minimize random effects, leading to a total of 9,720,000 simulation runs.

**Figure 2:**
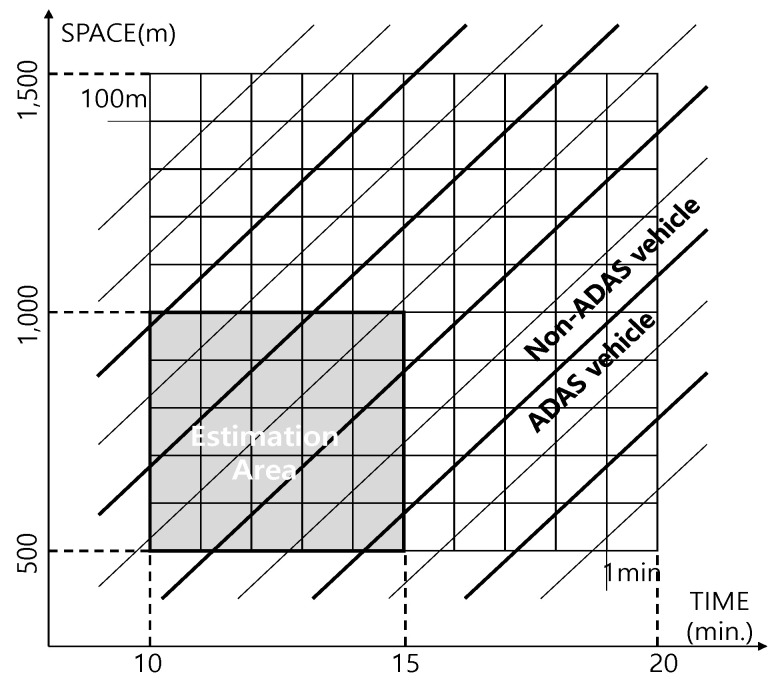
Concept of the estimation area with advanced driver assistance system (ADAS) vehicles (e.g., 300 s × 500 m area).

This study defines the estimation area spatially from 100 m to 1000 m in 100 m increments and temporally 60 s to 600 s in 60 s increments which leads to 100 cases. Although the traffic state can be estimated by observing vehicles for a longer period of time and along with the extended areas, when the spatial range is too long, various traffic characteristics are mixed so that the estimated traffic flow may not represent the actual traffic conditions of the network. Thus, this study set the maximum spatial boundary of the estimation area to 1000 m. Figure 2 shows 100 cases of estimation areas in time and space. The number of vehicles or their travel time and distance is different, even with the same traffic demand according to the defined estimation area. The Normalized Root Mean Square Error (NRMSE) can be used to standardize the error between groups with different units and was used to evaluate the accuracy of traffic state estimates (i.e., traffic volume, speed and density) for the individual scenarios. The true value of the traffic state for each scenario was obtained from the link evaluation output of the VISSIM simulation model.

The observed values ($q_{1},\;u_{1},\;k_{1})$ of traffic volume, speed and density are treated as constant values (q, u, k) derived from the link evaluation for each scenario without any nonrecurrent traffic states, and the average value of observed values ($\bar{q},\;\bar{u},\;\bar{k}$) is the same as (q, u, k). Based on traffic flow theory, NRMSE of the traffic volume has the same result as NRMSE of the density when the observed speed (u) and estimated speed ($\bar{u}$) are similar in (7).

(7) $$NRMSE\left(q\right)=\frac{\sqrt{\frac{\sum_{i=1}^{N}{\left(q_{i}-{\hat{q}}_{i}\right)}^{2}}{N}}}{\bar{q}}=\frac{\sqrt{\frac{\sum_{i=1}^{N}{\left(uk-{\hat{u}}_{i}{\hat{k}}_{i}\right)}^{2}}{N}}}{uk}=\frac{\sqrt{\frac{\sum_{i=1}^{N}u^{2}\left(k^{2}-2k{\hat{k}}_{i}+{\hat{k}}_{i}^{2}\right)}{N}}}{uk}=\frac{\sqrt{\frac{\sum_{i=1}^{N}{\left(k-{\hat{k}}_{i}\right)}^{2}}{N}}}{k}=NRMSE\left(k\right)$$

where:

N: number of samples
NRMSE(q): normalized root mean square errors of flow
NRMSE(k): normalized root mean square errors of density
q, u,k: true values of flow, speed and density
$q_{i},\;u_{i},\;k_{i}:\;$*i*th observed values of flow, speed and density
$\bar{q},\;\bar{u},\;\bar{k}:$ average values observed for flow, speed, and density
${\hat{q}}_{i},{\hat{u}}_{i},{\hat{k}}_{i}:$*i*th estimated values of flow, speed and density

**Figure 3:**
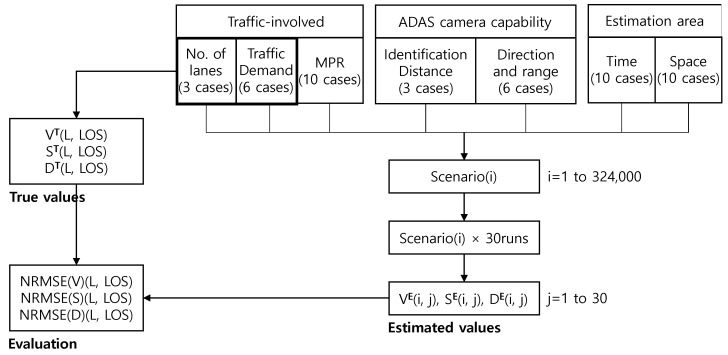
Traffic state estimation and evaluation framework.

Figure 3 illustrates the framework of traffic state estimation and evaluation concerning all relevant parameters, which was implemented using VISSIM and VISSIM COM. The total number of scenarios composed of seven parameters was 324,000 and 30 simulation runs for individual scenarios were implemented to estimate the traffic states. The estimated volume (V), speed (S) and density (D) were compared with the ground truth derived centered on the number of lanes and traffic demand to evaluate their accuracy with NRMSE. 

## 4. Experimental and Theoretical Distance Error of ADAS Camera

Dynamic error and the static error should be considered in the image-based vehicle identification technology using the ADAS camera. This study does not consider the dynamic error occurring from errors such as slope and bumps because there would be endless cases. The static error consists of parameter error, discretization error and calibration error. Since they occur in a regular pattern in the internal calculation process, it is possible to calculate them through an established formula.

The parameter error can be ignored if it occurs within the tolerance range and the other two errors can be calculated through Equations (8)–(13). As a result, the discretization error (11) and the calibration error (12) constituting the static error are directly related to the distance to the leading vehicle (10) [42]. The theoretical distance error corresponding to the distance to the leading vehicle was calculated based on the generally accepted values of the parameters for the static error estimation.

(8) $$y=\lambda \frac{h}{z}=ps\left(py-py_{0}\right)$$

(9) $$py-py_{0}=\lambda \frac{h}{ps\times z}$$

(10) $$z=\frac{\lambda h}{ps}\times \frac{1}{py-py_{0}}$$

(11) $${\left|\Delta z\right|}_{discretization}=\frac{\lambda h}{ps}\times \frac{1}{{\left(py-py_{0}\right)}^{2}}$$

(12) $${\left|\Delta z\right|}_{calibration}=\left(\frac{\lambda h}{ps}\times \frac{1}{{\left(py-py_{0}\right)}^{2}}\right)\left|\Delta py_{0}\right|$$

(13) $${\left|\Delta z\right|}_{static}={\left|\Delta z\right|}_{discretization}+{\left|\Delta z\right|}_{calibration}$$

where:

y: physical coordinate of the leading vehicle
z: distance to the leading vehicle
Δz: distance error
λ: nominal focal length=6.7 mm
yh: camera height=1.3 m
ps: pixel size=7.5 μm
y: pixel coordinate of the physical coordinate y
$py_{0}:\;location\;of\;vanishing\;point\;of\;\mathrm{py}=180$
py range: [190, 360]

**Figure 4:**
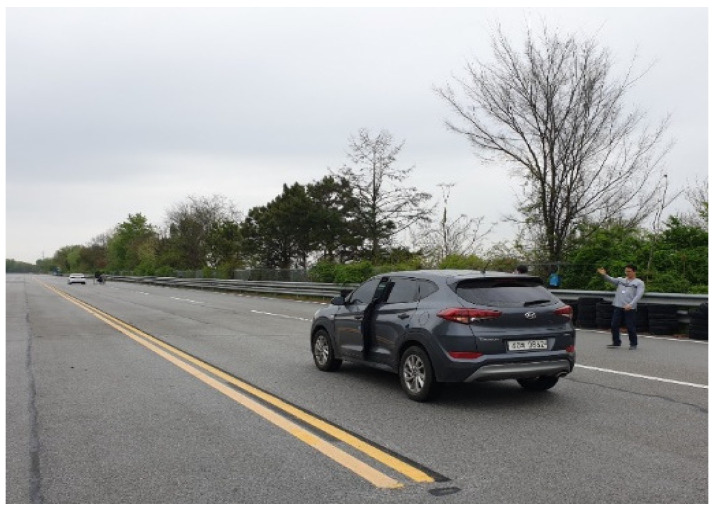
Field experiment test track.

**Figure 5:**
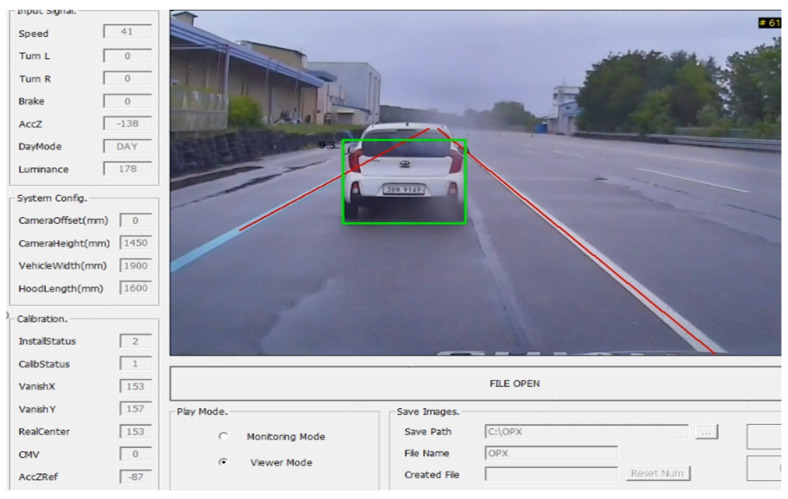
Field experiment for the image-based vehicle identification process.

In addition, a driving test was conducted to evaluate the performance of the ADAS camera on the test road of the Korea Automotive Technology Institute (KATECH). The road is located in Cheonan, Chungcheongnam-do, and the test was performed on 4 July 2019. Figure 4 and Figure 5 demonstrate the experimental process, where a high-speed vehicle equipped with radar and an ADAS camera approaches another vehicle moving at a constant speed. The same process was repeated 40 times, and the distance error of the ADAS camera was analyzed by comparing it with the actual distance from radar measurements. The initial distance between the subject vehicle (SV) and the principle other vehicle (POV) was set to 100 m in the test.

**Figure 6:**
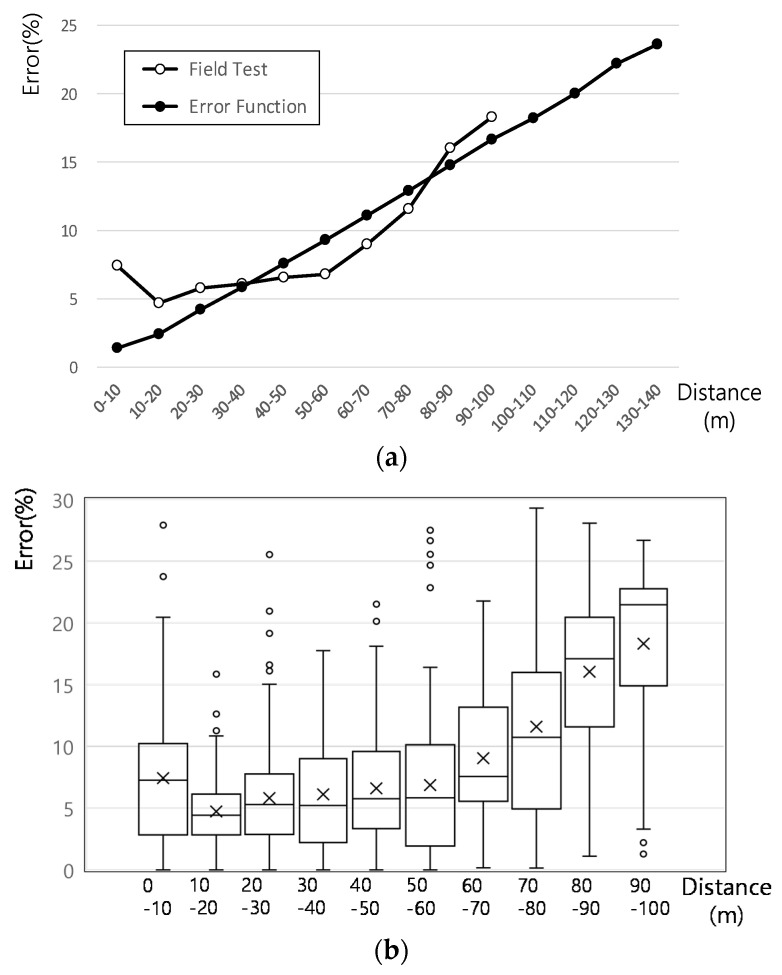
ADAS camera distance error: (**a**) Experimental and theoretical distance error comparison; (**b**) Variation of experimental distance error.

Figure 6 compares the experimental and theoretical mean distance error calculated from 0 m to the maximum identification distance of 140 m in 10 m intervals. The figure also shows the distribution of the distance estimated by the ADAS camera. At distances over 80 m, the experimental distance error was larger than the theoretical one, but the opposite result was shown from 30 m. However, the results showed similar tendencies. The reason for the error at 30 m or less is that the experiment was performed by abruptly changing lanes without reducing speed when the high-speed vehicle approaches the other vehicle. Therefore, the theoretical mean distance error at 10 m intervals was reflected as the maximum distance error corresponding to the headway between the ADAS vehicle and the leading vehicle in every simulation time to obtain a more realistic analysis result.

## 5. Simulation Analysis

The accuracy of the traffic volume, speed and density estimates was evaluated. The speed was estimated from the travel time and distance of the ADAS vehicle in the estimation area under recurrent traffic states, which proved to be a very stable estimate similar to the true value. Therefore, as shown in (7), NRMSE(*q*) and NRMSE(*k*) have almost identical values, and the analysis was conducted using only NRMSE(*k*).

**Figure 7:**
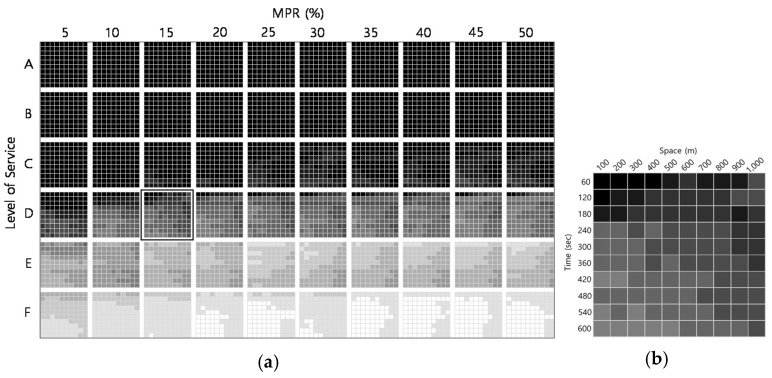
Normalized root mean square errors of density (NRMSE(*k*)): (**a**) One lane and identification distance 100 m case (i.e., 6000 cases); (**b**) One lane, distance 100 m, LOS D and MPR 15% case (i.e., 100 cases).

The estimated error for all numbers of lanes and vehicle identification distances is influenced by the LOS and MPR. Figure 7 shows the NRMSE(*k*) for one lane and the camera identification distance of 100 m based on the individual LOS and MPR. As the traffic demand and MPR increase, NRMSE(*k*) decreases because the low LOS and MPR cannot be used to identify the leading vehicle with a 100 m identification distance. Moreover, the estimated error of the specific combination of the LOS and MPR is affected by the spatiotemporal range of the estimation area. For example, the error for LOS D and MPR of 15% decreases at lower spatial and higher temporal resolution because of the driving behavior of the ADAS vehicle and the leading vehicle.

**Figure 8:**
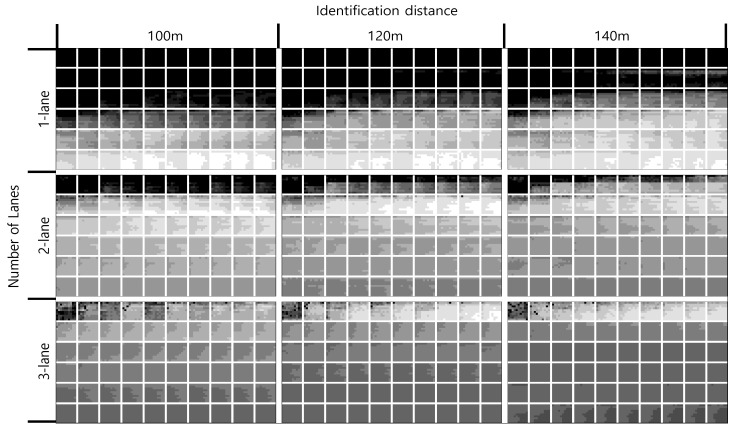
NRMSE(*k*) with front camera (i.e., 54,000 cases).

Contrary to expectations, there were few differences in NRMSE(*k*) depending on the camera direction and range. Figure 8 investigates the overall effect of the number of lanes and vehicle identification distance on NRMSE(*k*) with a front camera. As the number of lanes and the vehicle identification distance increase, the error of the traffic state estimates is improved, even at a lower LOS. Moreover, the error is remarkably improved with a lane increase from one to two lanes.

When the number of lanes and the vehicle identification distance increase, however, the estimated error deteriorates as the LOS increases. This happens because the density estimate can be recognized by identifying relatively closely located vehicles when the number of lanes is small and the vehicle identification distance is short. That is, the density estimate becomes larger than the true density. In contrast, as the vehicle identification distance and the number of lanes increase, it is possible to identify a relatively long distance from the leading vehicle that was not recognized in the previous situation, so the density estimate becomes smaller than the true density. Therefore, the improvement of the ADAS camera capabilities does not always ensure better accuracy of the estimates.

## 6. Conclusions

Traffic flow data, such as flow, density and speed, are used as the primary data for transportation planning and traffic operation. They are generally collected directly or estimated using stationary detectors with spatial limitations when acquiring detailed and wide-range traffic information simultaneously in an entire network. This study assessed ADAS cameras to help overcome the problem of stationary detectors in estimating the traffic states.

More realistic estimation results were derived than in previous studies by analyzing the experimental and theoretical distance error of an image-based vehicle identification method with an ADAS camera and reflecting it in a microscopic simulation model, VISSIM, and the accuracy of the estimates were evaluated. According to the results, the differences in NRMSE(*k*) with respect to the camera direction and range were small. Moreover, a long vehicle identification distance does not always ensure better accuracy of the estimates. This is because the density is estimated by identifying relatively closely located vehicles when the vehicle identification distance is short, which makes the estimate larger than the true density. As the vehicle identification distance increases, it is possible to recognize a relatively long distance from the leading vehicle, which enables the estimate to be less than the true density.

Any ADAS camera identification distance cannot overcome the distance limitation at LOS A, requiring long headway identification and resulting in very poor accuracy. However, the errors are significantly improved from LOS B. Moreover, the estimated error of the specific combination of LOS and MPR is affected by the spatiotemporal range of the estimation area as well. 

In conclusion, this study showed the feasibility of using an ADAS camera for traffic state estimation. However, the capability of the ADAS camera in relation to the camera direction and range does not significantly affect the accuracy of the traffic state estimates. Moreover, the vehicle identification distance does not always ensure better accuracy of the estimates. Therefore, it is recommended that transportation planners and traffic engineering practitioners carefully select various variables to secure a certain level of accuracy for traffic state estimates suitable for their purposes.

Considering the traffic management system used for implementing the contents of this study, the distance data can be transmitted to the traffic management center (TMC) through the vehicle-to-infrastructure (V2I) communication and the TMC can then estimate the traffic states and distribute them to the upstream vehicles through V2I. In future research, the trade-off between the accuracy of the traffic state estimates and the communication environment, such as V2I affected by the MPR, should be investigated.

## Figures and Tables

**Table 1:** Advantages and disadvantages of a traffic state estimation method based on a stationary detector and probe vehicle.

Type	Advantages	Disadvantages
Stationary detector	- possible to collect traffic data regardless of the time of day - used for various traffic operations and management	- spatially limited in data collection coverage - requires regular management and maintenance
Probe vehicle	- almost no spatial limitation in collecting data - additional equipment not required for collecting data	- less accurate without a sufficient number of probe vehicles - less feasible to estimate the interrupted traffic state

**Table 2:** Associated parameters and their ranges.

Category	Parameter	Number of Cases	Range
ADAS camera capability	Identification distance (m)	3	100, 120, 140
	Shooting direction and range	6	Front, rear, front and rear, all front, all rear, all front and all rear
Traffic involved	Number of lanes (lanes)	3	1, 2, 3
	Traffic demand (vph)	6	LOS A to LOS F
	MPR (%)	10	5 to 50 in 5% increment
Estimation area	Time (s)	10	60 to 600 in 60-s increment
	Space (m)	10	100 to 1000 in 100 m increment
Simulation	Multiple runs (times)	30	30 different random numbers
Total number of scenarios		324,000	
Total number of simulation runs (times)		9,720,000	

## Data Availability

Not applicable.
